# 4‑Chlorobenzylamine Containing Maleic Acid Derivatives: Synthesis, *In Silico* Studies, and Anti-Alzheimer’s Activity

**DOI:** 10.1021/acschemneuro.5c00449

**Published:** 2026-02-03

**Authors:** Muhammad Junaid Tariq, Madiha Kanwal, Athar Ata, Humaira Nadeem, Mahwish Siddiqui

**Affiliations:** † Department of Pharmaceutical Chemistry, Riphah Institute of Pharmaceutical Sciences, 66783 Riphah International University, Islamabad 44000, Pakistan; ‡ 439597 Capital University of Science and Technology, Islamabad 44000, Pakistan; § Department of Chemistry, Michigan Technological University, Houghton, Michigan 49931-1295, United States

**Keywords:** maleic acid derivatives, Alzheimer, DPPH assay, ACE assay, docking, IHC, H&E

## Abstract

One way to protect neurons is to protect them from oxidative damage by reducing lipid peroxidation (LPO). Therapeutic medicines that target the inflammatory response have antioxidant activities and can also block inflammatory cascade pathways and counteract cell lyses. The goal of this investigation was to see if new maleic acid derivatives could protect the brain from scopolamine-induced amnesia. To evaluate and characterize the maleic acid derivatives, spectroscopic techniques such as ^1^H NMR and Fourier Transform Infrared Spectroscopy (FTIR) were used. To further evaluate the synthesized compounds, an in vitro DPPH antioxidant assay was performed, compound 2f exhibited the best antioxidant potential, and along this side, an acetylcholinesterase (ACE) inhibition assay was performed. Compounds 2a and 2f showed promising results with IC_50_ 20.15 and 22.09 nM, respectively. Scopolamine-treated rats trigger neurodegeneration, raise the level of antioxidant enzymes, and increase oxidative stress. The elevated levels of Tumor Necrosis Factor α (TNF-α), cyclooxygenase-2 (COX-2), Jun N-terminal kinase (JNK), and Nuclear Factor kappa-light-chain-enhancer of activated B cells (NFκB), which are neuroinflammatory mediators, along with neuronal damage, were also seen. The anti-Alzheimer’s activity of maleic acid derivatives was performed in these rats by performing the Y-maze test, Morris water maze (MWM) models, immunohistochemistry, and hematoxylin and eosin staining. In vivo antioxidant assays revealed that compounds 2a and 2f significantly restored enzymatic defenses and reduced lipid peroxidation, with 2a showing slightly superior activity. The maleic acid derivatives (2a and 2f) cause increased spontaneous changes in the rat behavior and the number of entries of rats in the Y-maze test. The observation from the MWM model showed a decrease in the escape latency time in the rats. Finally, the AutoDock Vina program was used to check ligand-protein interaction using COX-2, and TNF-α, JNK, NFκB, GSK-3β, and ACE were used as targets.

## Graphical abstract

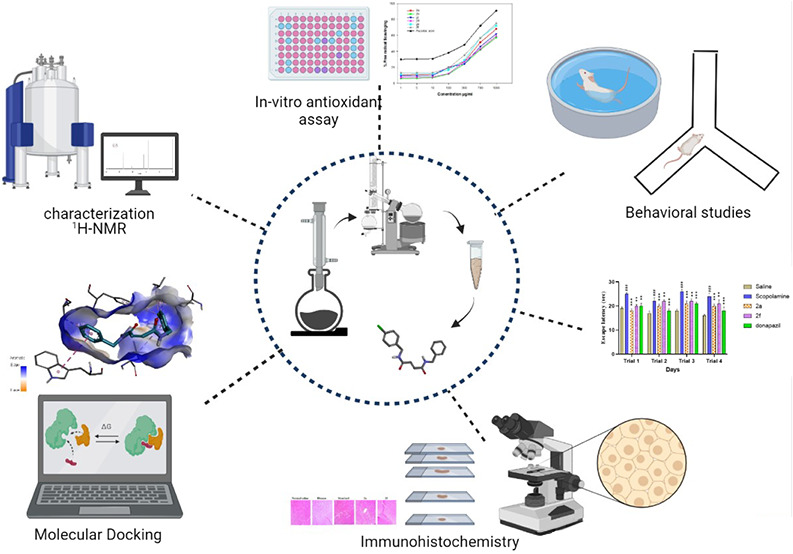

## 1 Introduction

Neurodegeneration is the irreversible damage to the brain and includes various malfunctions in the CNS functionalities and diseases such as Alzheimer’s, which is the most common cause of dementia and memory impairment, ataxia, which is abnormality in muscles coordination, Parkinson’s disease, which causes trembling and failure in muscles coordination, Huntington’ beings disease, which causes uncontrolled movement of muscles and distorted thinking ability, and various other diseases.^1–4^ In recent years, over 50 million individuals were affected by Alzheimer’s disease (AD), and it is predicted that the progression of AD will double its number by the year 2050.^5^ Neurodegenerative diseases such as AD are the root cause of progressive memory impairment, which leads to dementia, and it is one of the prime concerns in the field of medical science.^6^ As the human body is unable to regrow and reproduce neurons after the completion of the fetal stage, the increase in the number of neurons halts, and the development of neurons is hypertrophic not hyperplastic.^7,8^ Various therapeutic approaches were observed for the treatment of neurodegenerative disorders, and various compounds are in the pipeline to cure AD,^9–13^ but the discovery of a treatment for AD is not only tedious but also very exorbitant and strenuous.^14,15^ After years of research, no such treatment for AD is ascertained, which can reverse the damage done to the neurons in AD. The unavailability of a cure for AD makes it a potentially important target for drug discovery.

Oxidative stress, which is the imbalance between the reactive oxygen species and natural antioxidants produced by the body to capture them, causes excess of oxidants. It causes the differential expression of various genes that regulate the production of inflammatory mediators.^16^ Recent reports recapitulated that oxidative stress and inflammatory mediators are major contributors to cerebral insult.^17–19^ The overproduction of inflammatory mediators and reactive oxygen species causes the accumulation of deformed proteins such as amyloid β plaques and tau protein.^20–22^ Furthermore, hyperactivation of astrocytes and microglial cells causes the overproduction of reactive oxygen species (ROS).^23^ Moreover, the generation of numerous inflammatory mediators causes dysfunction of microtubules and mitochondria.^24,25^ The overproduction of inflammatory mediators causes disruption of normal physiological functions of cells, leading to cellular injury and apoptosis.^26^ Certain molecules that play a significant role in specific areas of the body when overexpressed cause harm and damage to normal cellular function. The conventional inflammatory mediators such as COX-2, TNF-α, NF-κB, and JNK are the key indicator inflammatory molecules that play several protective roles in the body but, when overexpressed due to the accumulation of amyloid β proteins, become key molecules to assist the progression of AD.^27,28^ It has been reported that COX-2 is one of the major factors which play a significant role in the inflammatory cascade and progression of AD. Overproduction of COX-2 causes cellular inflammation and dysfunction of the physiological activity of the cell.^29–31^ Likewise, TNF-α is also a major contributor to the progression of AD, and blocking the TNF-α pathway has shown improvement in the cognitive functions of the brain in various studies.^32–34^ Similarly, nuclear factor (NFκB) is an indicator of AD. Overproduction of NFκB leads to aging and increased COX-2 and reactive oxygen species which leads to neurodegeneration.^35^ Through research, it is evident that a certain family of signaling proteins, JNKs, which are key molecules to play a role in apoptosis, are associated with neurodegeneration. JNKs are a family of protein kinases that play a role in gene expression, neuronal plasticity, regulation of cellular senses, regeneration, and cell death. Due to various irregularities such as oxidative stress and amyloid protein accumulation, the JNK pathway is activated and causes cellular damage which includes neurodegeneration and AD.^36,37^

Apart from the fact that inflammatory mediators are the key factors in neurodegeneration, it is also well-established that a low level of muscarinic agonists like acetylcholine or an increased amount of muscarinic antagonists such as scopolamine causes memory impairment and dementia.^38^ The activity of scopolamine is related to acetylcholine esterase (ACE) activity, which alters the levels of reactive oxygen species. The imbalance of ROS and natural antioxidant glutathione causes oxidative stress causing an increase in the level of inflammatory mediators such as COX-2 and TNF-α.^39–41^ Through experimental research, it is well established that certain types of acetylcholine esterase inhibitors which can cross the blood–brain barrier (BBB) are very effective in counteracting the neurodegeneration that causes dementia,^42^ as shown in Figure 1.

**Figure 1:**
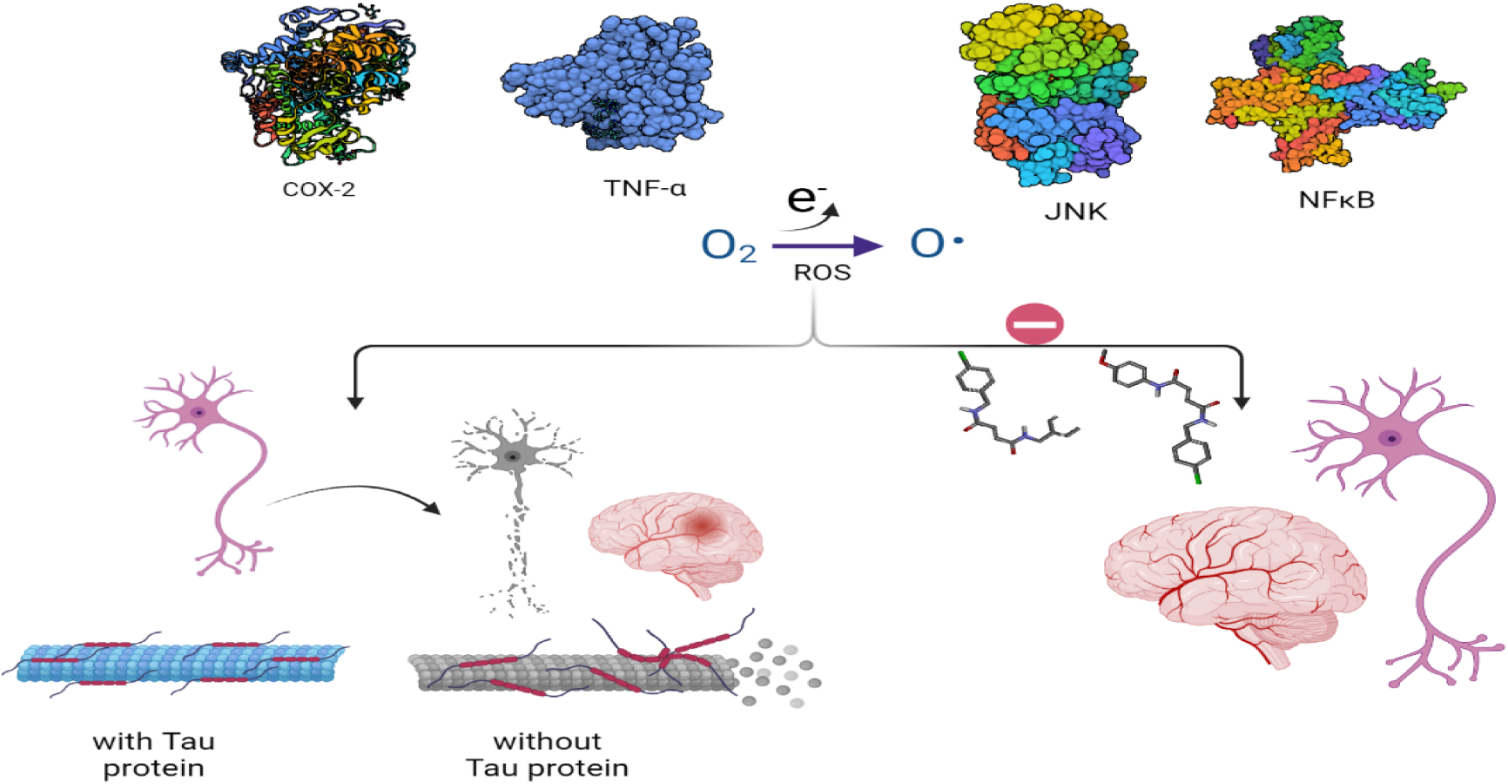
Damage to neurons by reactive oxygen species is prevented by blocking the pathway of inflammatory mediators.

In recent studies, numerous compounds have shown the effect of reducing these pivotal causes of AD. Like ACE inhibitors, Nonsteroidal Anti-Inflammatory Drug (NSAID) is also one of the classes of drugs that restrains the progression of AD.^26^ NSAIDs block the activity of COX-2 enzyme receptors and play a role in reducing the progression of AD. Compounds such as amides are very useful in a variety of diseases and infections. Amides show antibacterial,^43^ antifungal,^44^ antiviral,^45^ and various other activities. Succinimide derivatives show increased anti-Alzheimer activity by blocking the pathway of the inflammatory cascade.^46^

Amide derivatives are very potential candidates in various fields of medicine.^47^ Amide bonding in the molecule is the imperative functionality that makes the molecule very lucrative. In this study, some novel maleic acid derivatives were designed and synthesized. The usefulness in the field of neuroprotection was taken into account. It is considered whether these compounds are useful for future research.

## 2 Materials and Methods

### 2.1 Chemicals and Solvents

Solvents used in this study were purchased from Sigma-Aldrich, Merck, and Honeywell. Solvents were distilled before use. Dichloromethane was freshly distilled over calcium hydride prior to use. The reactions were carried out using dried glassware. Dichloromethane, 4-chlorobenzyl amine, maleic anhydride, thionyl chloride, and triethylamine of analytical grade were used during the synthesis.

### 2.2 The General Procedure of Synthesis of 4-((4-Chlorobenzyl)­amino) 4-Oxobut-2-enoic Acid

Equimolar quantities of 4-chlorobenzylamine and maleic anhydride were dissolved in dichloromethane. The mixture was stirred for 20 min. Completion of the reaction was checked by Thin Layer Chromatography (TLC). After the completion of the reaction, the solid was separated by filtration, and further recrystallization with methanol was done to obtain further purity.^48^

### 2.3 General Procedure for the Synthesis of Amide Derivatives 2­(a–f)

One mmol of 4-((4-chlorobenzyl) amino) 4-oxobut-2-enoic acid, 1 mmol of respective amine, and 3 mmol of triethylamine were added to dichloromethane, followed by the addition of 1 mmol of thionyl chloride (SOCl_2_) to the reaction mixture. The mixture was stirred for about an hour at room temperature.

After the completion of the reaction, as checked by the TLC, the solvent was evaporated by a rotary evaporator. The resultant residue was dissolved in dichloromethane and then washed with 1 N HCl first and with 1 N NaOH afterward. The organic layer was separated and dried over anhydrous sodium sulfate (NaSO_4_) and filtered. Evaporation of the solvent provided the required amide derivative 2 (a–f) (Figure 2).

**Figure 2:**
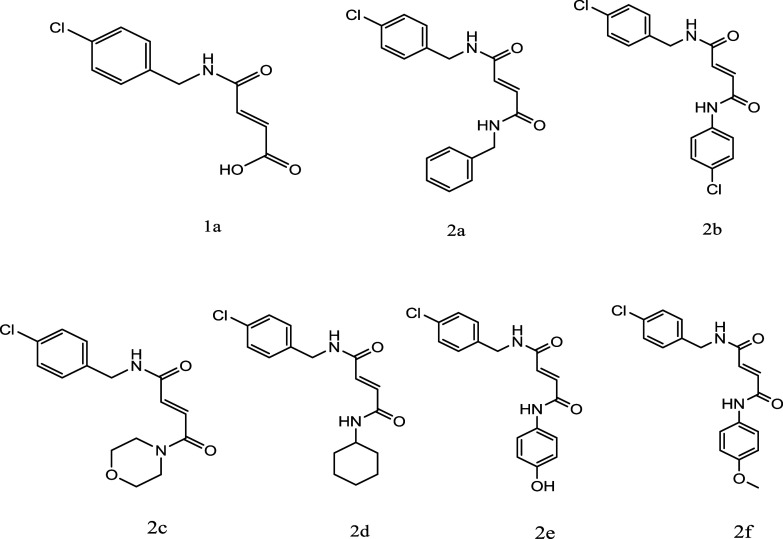
Chemical structure of the newly synthesized compounds.

#### 2.3.1 4-((4-Chlorobenzyl)­amino) 4-Oxobut-2-enoic Acid (1a)

FTIR (υ̅, cm^–1^): 1693 Carboxylic (C=O), 1488 (C=C), 3224 (N–H): ^1^H NMR (DMSO-*d6*, 300 MHz, δ ppm): 3.61 (s, 2H, CH_2_), 6.85 (d, H, *J* = 7.8 Hz, CH), 6.95 (d, H, *J* = 7.8 Hz, CH), 7.35 (d, 2H, *J* = 6.2 Hz, Aryl-H), 7.56 (d, 2H, *J* = 7.6 Hz, Aryl-H), 9.73 (s, 1H, Amide-H). ^13^C NMR: δ 40.2 (1C, s), 128.5 (2C, s), 129.4 (2C, s), 132.0 (1C, s), 133.3 (1C, s), 134.4 (1C, s), 135.3 (1C, s), 161.9 (1C, s), 167.0 (1C, s).

#### 2.3.2 N1-Benzyl-N4-(4-chlorobenzyl) Fumaramide (2a)

FTIR (υ̅, cm^–1^): 1670 (C=O), 1523 (C=C), 3342 (N–H): ^1^H NMR (DMSO-*d6*, 300 MHz, δ ppm): 3.61 (s, 2H, CH_2_), 6.97 (d, 1H, *J* = 7.2 Hz, CH), 7.10 (d, 1H, *J* = 6.8 Hz, CH), 7.26–7.35 (m, 5H, Aryl-H), 7.35 (d, 2H, *J* = 6.7 Hz, Aryl-H), 7.56 (d, 2H, *J* = 7.4, Aryl-H), 9.73 (s, H, Amide-NH). ^13^C NMR: δ 40.1–42.2 (2C, 42.2 (s), 42.2 (s)), 116.09 (1C, s), 127.2–127.3 (3C, 127.2 (s), 127.2 (s)), 128.3 (2C, s), 128.5 (2C, s), 129.4 (2C, s), 133.3 (1C, s), 134.4 (1C, s), 135.2–135.3 (2C, 135.3 (s), 135.3 (s)), 138.9 (1C, s), 166.9–167.1 (2C, 161.0 (s), 161.0 (s)).

#### 2.3.3 N1-(4-Chlorobenzyl)-N4-(4-chlorophenyl) Fumaramide (2b)

FTIR (υ̅, cm^–1^): 1665 (C=O), 1530 (C=C), 3356 (N–H): ^1^H NMR (DMSO-*d6*, 300 MHz, δ ppm): 3.62 (s, 2H, CH_2_), 6.96 (d, 1H, *J* = 7.0 Hz, CH), 7.12 (d, 1H, *J* = 6.8 Hz, CH), 7.5–7.71 (m, 4H, Aryl-H), 7.51 (d, 2H, Aryl-H), 9.36 (s, 1H, Amide-NH). ^13^C NMR: δ 40.2 (1C, s), 121.6 (2C, s), 127.0 (1C, s), 128.5 (2C, s), 129.0 (2C, s), 129.4 (2C, s), 133.3 (1C, s), 134.4 (1C, s), 135.2–135.3 (2C, 135.3 (s), 135.3 (s), 140.2 (1C, s), 164.7 (1C, s), 167.0 (1C, s).

#### 2.3.4 N-(4-Chlorobenzyl)-4-morpholino-4-oxobut-2-enamide (2c)

FTIR (υ̅, cm^–1^): 1671 (C=O), 1528 (C=C), 3360 (N–H): ^1^H NMR (DMSO-*d6*, 300 MHz, δ ppm): 3.33–3.52 (m, 8H, Morpholine-H), 3.61 (s, 2H, CH_2_), 6.67 (d, 1H, *J* = 7.1 Hz, CH), 7.14 (d, 1H, *J* = 6.81 Hz, CH), 7.35 (d, 2H, *J* = 6.5 Hz, Aryl-H), 7.56 (d, 2H, *J =* 7.0 Hz, Aryl-H), 9.36 (s, 2H, Amide-H). ^13^C NMR: δ 40.0 (2C, s), 42.2 (1C, s), 66.5 (2C, s), 121.9 (1C, s), 128.5 (2C, s), 129.4 (2C, s), 133.3 (1C, s), 134.4 (1C, s), 135.3 (1C, s), 164.4 (1C, s), 167.0 (1C, s).

#### 2.3.5 N1-(4-Chlorobenzyl)-N4-cyclohexylfumaramide (2d)

FTIR (υ̅, cm^–1^): 1675 (C=O), 1532 (C=C), 3354 (N–H) ^1^H NMR (DMSO-*d6*, 300 MHz, δ ppm): 1.11–1.74 (m, 10H, Cyclohexyl-H), 4.54 (m, 1H, CH), 3.65 (s, 1H, CH_2_), 6.66 (d, 1H, *J* = 7.6 Hz, CH), 7.2 (d, 1H, *J* = 6.8 Hz, CH), 7.32 (d, 2H, H, *J* = 6.8 Hz, Aryl-H), 7.55 (d, 2H, Amide-NH), 9.36 (s,1H, Amide-H). ^13^C NMR: δ 24.8 (2C, s), 25.7 (1C, s), 32.9 (2C, s), 42.2 (1C, s), 48.6 (1C, s), 128.5 (2C, s), 129.4 (2C, s), 133.3 (1C, s), 134.4 (1C, s), 135.2–135.3 (2C, 135.3 (s), 135.3 (s), 167.0 (1C, s), 170.1 (1C, s).

#### 2.3.6 N1-(4-Chlorobenzyl)-N4-(4-hydroxyphenyl)­fumaramide (2e)

FTIR (υ̅, cm^–1^): 1678 (C=O), 1537 (C=C), 3345 (N–H): ^1^H NMR (DMSO-*d6*,300 MHz, δ ppm): 4.2 (s, 2H, CH_2_), 6.62 (d, H, *J* = 7.6 Hz, CH), 7.11 (d, 1H, *J* = 6.7 Hz, CH), 7.2, 7.45 (m, 8H, Aryl-H), 11.48 (s, 1H, Amide-H). ^13^C NMR: δ 40.2 (1C, s), 115.2 (2C, s), 120.9 (2C, s), 128.5 (2C, s), 129.4 (2C, s), 133.3 (1C, s), 134.4 (1C, s), 134.8 (1C, s), 135.2–135.3 (2C, 135.3 (s), 135.3 (s), 152.8 (1C, s), 164.7 (1C, s), 167.0 (1C, s).

#### 2.3.7 N1-(4-Chlorobenzyl)-N4-(4-methoxyphenyl) Fumaramide (2f)

FTIR (υ̅, cm^–1^): 1667 (C=O), 1540 (C=C), 3360 (N–H): ^1^H NMR (DMSO-*d6*, 300 MHz, δ ppm): 3.82 (s, 3H, OCH_3_), 4.35 (s, 2H, CH_2_), 6.7 (d, 1H, *J* = 7.4 Hz, CH), 7.41 (d, 1H, *J* = 6.6 Hz, CH), 6.90–77 (m, 8H, Aryl-H), 9.36 (s, 2H, Amides-H). ^13^C NMR: δ 40.1–42.2 (2C, 42.2 (s), 42.2 (s)), 55.3 (1C, s), 113.8 (2C, s), 128.3–128.6 (4C, 128.4 (s), 128.5 (s)), 129.4 (2C, s), 133.3 (1C, s), 134.4–134.5 (2C, 134.4 (s), 134.4 (s)), 135.2–135.3 (2C, 135.3 (s), 135.3 (s)), 159.0 (1C, s), 166.9–167.1 (2C, 160.0 (s), 160.0 (s)).

### 2.4 Purification

Purification of the synthesized compounds was done by recrystallization. Synthesis and purity of the compound are checked by performing thin layer chromatography by using the following solvent system: (Chloroform:methanol 2:1), (Ethyl acetate:petroleum ether 1:2). To perform TLC, Merck TLC silica gel F254 precoated plates were used. Visualization of the spot is done by using a UV lamp (254 nm).

### 2.5 Characterization

Characterization of newly synthesized compounds was carried out by using a Bruker ALPHA FTIR spectrometer with Eco ATR; ^1^HNMR and ^13^C NMR were recorded by using a Bruker AM300 spectrophotometer with chloroform-d as solvent.

### 2.6 Animals

Animals used: Adult Sprague–Dawley rats of either sex, weighing between 95 and 105 g, were housed in controlled temperature between 22 and 25 °C. A light and dark cycle of 12 h each was provided to the animal. Free access to food and water was provided. Animals were randomly divided into groups. One is treated with saline, 2 with the test compounds, one reference (scopolamine), and one standard treatment group. By using AD models, behavioral studies were performed to investigate the anti-Alzheimer effect of the synthesized compounds. The animal studies were done according to the rulings of the Institute of Laboratory Animal Resource, Commission on Life Sciences, University, and National Research Council . We affirm that all methods employed in this undertaking were conducted in strict adherence to the pertinent guidelines and regulations governing the respective field, ensuring the integrity, ethical standards, and compliance with established protocols throughout the entire process, and we also confirm that all methodologies employed in this study are reported in strict accordance with the ARRIVE (Animal Research: Reporting of In Vivo Experiments) guidelines, ensuring comprehensive and transparent documentation of our experimental procedures for the benefit of scientific rigor and reproducibility. During experimentation, all protocols and procedures were carried out as per the guidelines approved by Research and Ethical Committee (REC) of Riphah International University (Approval ID: Ref. No. REC/RIPS/2021/25).

#### 2.6.1 Y-Maze Test

The Y-maze test is used to test spatial working memory. The Y maze is a 3-armed horizontal maze with the following dimensions: 50 cm in length, 10 cm in width, and walls 20 cm in height. The arms are separated at an equal distance of 120° from each other. Rats were divided into 5 groups having five rats in each group (*n* = 5). Each group was administered a single dose per day for 4 days. Group I was taken as the negative control and was administered an intraperitoneal dose of normal saline (10 mL/kg). Group II was taken as the disease group and was administered an intraperitoneal dose of scopolamine (3 mg/kg).^49^ While groups III and IV (Disease + treatment), rats were treated with 10 mg/kg of the test compound intraperitoneally 1 h^50^ before the test, and after 30 min, memory impairment was induced by the administration of scopolamine (3 mg/kg) intraperitoneally. Several structurally or pharmacologically similar compounds have demonstrated efficacy around this dose.^50–52^ Group V was taken as the positive control and was administered an intraperitoneal dose of Donepezil (3 mg/kg).^53^ The arms of the Y-maze were thoroughly cleaned with 5% ethanol after every test to remove the odor and waste. To determine the percentage of alternation, the following equation was used.^54^

%Alternation=[(Numberofalternations)/Totalarmentries‐2)]×100

#### 2.6.2 Morris Water Maze Test

Rat groups aligned into (*n* = 5) which were treated in the Y-maze test are then exposed to swimming training for 60 s in the absence of a platform on day one. Now for the rest of four consecutive days, the rats were provided with a platform in the swimming area. Once the rat located the platform, it was allowed to remain on the platform for 10 s before being removed from the pool. If the rat was unable to locate the platform within 120 s, it was placed on the platform for 10 s and then removed from the pool. One day after the last training trial session, each mouse was individually subjected to a probe trial session in which the platform was removed from the pool. The mouse was allowed to swim for 120 s, and escape latency time was recorded by a video camera. A decrease in escape latency time showed an anti-Alzheimer potential.

### 2.7 Hematoxylin–Eosin (H&E) Staining

Hematoxylin–eosin (H&E) staining is used to visualize the pathology of tissues. In this staining technique, first tissue slides were prepared and deparaffinized with absolute xylene (100%), then rehydrated using a gradient ethanol series (100% to 70%), and finally washed with distilled water.^55^ Now the prepared slides were dipped in hematoxylin for 10 min, kept on an oscillator for 5 min in water, and finally treated with 1% HCl and 1% ammonia-water. Then the slides were dipped in the eosin solution for 5–10 min, rinsed with water, and air-dried. Dried slides were then dehydrated using graded ethanol (70%, 95%, and 100%) and mounted on glass coverslips. Pictures were taken by using an Olympus light microscope, and the images were analyzed for histological examination.

### 2.8 Immunohistochemical Analysis

Immunohistochemical staining was performed with little modification compared to Hematoxylin and Eosin (H&E) staining. Deparaffinization of slides was done with absolute xylene and a gradient ethanol series, followed by treatment with proteinase K which is an enzymatic process for the retrieval of antigen, and then washed with 0.1 M phosphate-buffered saline (PBS). 4% Hydrogen peroxide (H_2_O_2_) solution in methanol was applied to block the peroxidase activity. After some time, the slides were washed with phosphate-buffered saline (PBS), 3–5% goat serum was applied, and then incubated with 0.1% Tritn X-100 and primary antibodies for the whole night. Primary antibodies included JNK, TNF-α, COX-2, NFκB, and TRX (dilution 1:100, Santa Cruz Biotechnology). On the next day, slides were again washed with 0.1 M (PBS), incubated with secondary antibody biotinylated tagged (dilution 1:50), and finally with the ABC Elite kit (Santa Cruz Biotechnology) for 1 h in a humidified chamber. The slides were again washed with 0.1 M PBS, stained in 3,3′-diaminobenzidine (DAB) solution, and fixed using a graded ethanol series, followed by xylene, and then coverslipped. Immunohistochemical tagged image file format (TIFF) images were taken with a light microscope. Image software was used to quantitatively determine TNF-α and COX-2 by optimizing the background of images according to threshold intensity and analyzing the positive cells at the threshold intensity for all groups. The intensity was expressed as the relative integrated density of the samples relative to the saline.

### 2.9 Free Radical Scavenging Activity of Synthesized Maleic Acid Derivatives by the DPPH Method

Free radicals are key molecules responsible for the disruption of cellular functions, especially in neurons, and the progression of neurodegenerative diseases such as Alzheimer’s is the result of increased free radicals in the body. To check the antioxidant activity of synthesized maleic acid derivatives, a 2,2-diphenyl-1-picrylhydrazyl (DPPH) free radical scavenging assay was performed. A 1 mg/mL stock solution of test chemicals and ascorbic acid (positive control) was created, and subsequent sample concentrations (700, 300, 100, 10, 5, and 1 μg/mL) were prepared using the serial dilution method. Methanol was used to make the 1 mmol of DPPH solution. Then, from each dilution, 1 mL of the test sample was obtained, and 3 mL of DPPH solution was placed in separate test tubes to make a volume of up to 4 mL in each test tube. All test tubes were stored at room temperature and covered with aluminum foil. If test chemicals have oxidation potential, the naturally purple color of DPPH will turn yellow due to the free radical scavenging activity. Using a UV spectrophotometer set to 517 nm, the absorbance was measured. The inhibition percentage of free radical scavenging activity was calculated by using the formula as under.

$$\%\mathrm{Scavenging}\;\mathrm{activity}=\frac{\mathrm{Absorbance}\;\mathrm{of}\;\mathrm{control}-\mathrm{Absorbance}\;\mathrm{of}\;\mathrm{sample}}{\mathrm{Absorbance}\;\mathrm{of}\;\mathrm{control}}\times 100$$

### 2.10 Acetylcholinesterase (ACE) Inhibition Assay

The experiments were performed using a colorimetric ACE screening kit (BioVision, Cat. No. K197-100, USA) in a 96-well plate format. Stock solutions of each compound were prepared in assay buffer and serially diluted to obtain 20× working concentrations. From each working solution, 10 μL was transferred to the designated wells, followed by the addition of the enzyme, substrate (acetylthiocholine iodide, ATChI), and chromogenic reagent 5,5′-dithiobis­(2-nitrobenzoic acid, DTNB). The mixtures were incubated for 30 min at room temperature, and the absorbance was measured at 412 nm using a microplate reader.^56^ Donepezil served as the standard reference inhibitor, while control wells containing the enzyme and buffer without inhibitors were used to determine baseline activity. The degree of ACE inhibition was calculated by comparing treated wells to the control.

### 2.11 Chemo-Informatics

Software used to get cheminformatics was Chem Sketch, and online freeware tools were used to analyze chemo-informatics of synthesized compounds. The different parameters calculated and reported include molecular formula, LogP values, hydrogen bond donors, Polar Surface Area (PSA), molecular weight, and Lipinski rule validation. The structure of the single molecule assumed from the Nuclear Magnetic Resonance (NMR) is uploaded online, which generates an output in the form of the aforementioned parameters .

### 2.12 Molecular Docking

Docking of all the molecules was performed against a specific protein, and the effects were recorded. Molecular docking studies were performed using free license software. The computer used for molecular docking was HP Laptop Intel CORE i5 vPro. Different parameters and cheminformatics are measured for the synthesized compounds by using chemoinformatics. Studio Visualizer v17.2.0.16349 and AutoDock Tool v1.5.6, were the software used to find ligand and protein interactions and their binding affinities. Auto Dock Vina 1.1 was used for molecular docking.

#### 2.12.1 Preparation of Target Protein

Docking studies were performed by docking maleic acid derivatives (2a–2f) against COX-2, TNF-α, JNK, and NFκB. The relative binding affinities of ligands and proteins were recorded and compared. Protein structures (PDB: 5FI9, 5MU8, 2G01, and 1SVC), respectively, were downloaded from RCSB from the protein data bank (PDB) site. Discovery Studio was used to prepare ligand molecules by removing water molecules and heteroatoms and to obtain clean molecules with polar hydrogens.

#### 2.12.2 Ligand Preparation

All ligands were drawn individually in Discovery Studio in 3D form and saved in the PDB format. Then, the ligand was saved for docking in PDBQT format using the AutoDock tool.

#### 2.12.3 Docking Analysis and Visualization of Binding Conformations

Pyrex software was used for the selection of the grid box. Then the best molecular conformation that has the best binding energies (kcal/mol) was selected. A Discovery Studio Visualizer was used to visualize the binding conformation with the lowest energy coefficients. Then by using spatial (3D) and linear (2D) interaction maps, the ligand interaction with protein amino acids was visualized and recorded.

### 2.13 Evaluation of Antioxidant Potential

#### 2.13.1 Glutathione (GSH) and Glutathione-S-Transferase (GST) Assay

Glutathione (GSH) and glutathione-S-transferase (GST) levels were determined as biomarkers of oxidative stress by using previously described methods with minor modifications. Brain tissues were homogenized in phosphate buffer and treated with phenylmethylsulfonyl fluoride (PMSF), followed by centrifugation. The supernatant was collected, and the GSH content was quantified using 5,5′-dithiobis­(2-nitrobenzoic acid) (DTNB) as the reagent. Absorbance was measured at 412 nm after 15 min, and results were expressed as μmol/mg of protein. For GST activity, the assay mixture consisted of phosphate buffer (pH 6.5), GSH, and CDNB, to which the tissue supernatant was added. The conjugate formation was monitored at 340 nm using a microplate reader.^56^ All assays were carried out in triplicate, and the mean values were calculated from five independent samples (*n* = 5).

#### 2.13.2 Lipid Peroxidation Assay

Lipid peroxidation was assessed as a marker of oxidative stress by estimating thiobarbituric acid reactive substances (TBARS) following a modified colorimetric procedure.^56^ In brief, 200 μL of tissue supernatant was mixed with 200 μL of 100 mM ascorbic acid, 580 μL of 0.1 M phosphate buffer (pH 7.4), and 20 μL of ferric chloride solution, and the mixture was incubated at 37 °C for 1 h in a water bath. The reaction was terminated by adding 1 mL of 10% trichloroacetic acid (TCA) and 1 mL of 0.66% thiobarbituric acid (TBA), followed by incubation in a boiling water bath for 20 min with rapid cooling in ice water. Samples were then centrifuged for 10 min, and the absorbance of the supernatant was recorded at 535 nm. The extent of lipid peroxidation was expressed as nanomoles of malondialdehyde (MDA) formed per minute per milligram of protein.

#### 2.13.3 Catalase Assay

Catalase activity was determined using a previously reported protocol with minor modifications.^56^ Briefly, 10 μL of the sample was mixed with 290 μL of 3% hydrogen peroxide (H_2_O_2_) in each well, and the decomposition of H_2_O_2_ was monitored by measuring absorbance at 440 nm using a spectrophotometer

## 3 Results and Discussion

The maleic acid derivatives were synthesized by using the scheme shown in Figure 3.

**Figure 3:**
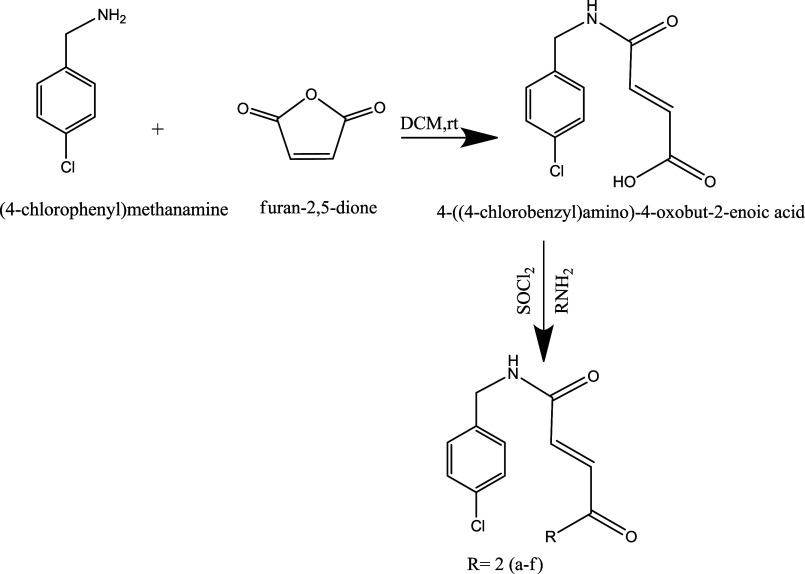
Scheme of the reaction to synthesize the compounds.

The purity of the compound was checked by TLC, and the characterization was done by ^1^H NMR. Purification and recrystallization of synthesized compounds were done, compound 1 was an amorphous solid, and compounds 2a–2f are sticky semisolids in nature having color in the range of pale yellow to brown % yield of the compound ranges from 35 to 85%. All molecules were assessed and fulfilled the Lipinski rule criteria. Further cheminformatics and physical properties are given in Tables 1 and 2.

**Table 1:** Physical Properties of Synthesized Compounds

Compounds	Molecular formula	Molecular weight(g/mol)	Physical state	Solubility	Color	Percentage yield
1	C_11_H_10_ClNO_3_	239.65	Solid	Methanol	White	85%
2a	C_17_H_15_ClN_2_O_2_	314.76	Semisolid	Methanol	Brown	35%
2b	C_17_H_14_Cl_2_N_2_O_2_	349.21	Semisolid	Methanol	Dark brown	46%
2c	C_15_H_17_ClN_2_O_3_	308.76	Semisolid	Methanol	Pale yellow	65%
2d	C_17_H_21_ClN_2_O_2_	320.81	Semisolid	Methanol	Brown	40%
2e	C_17_H_16_N_2_O_3_	296.32	Semisolid	Methanol	Brown	55%
2f	C_18_H_17_ClN_2_O_3_	344.79	Semisolid	Methanol	Dark brown	60%

**Table 2:** Chemo-Informatics of Synthesized Compounds

Compounds	Molecular volume (cm^3^)	Polarizability (cm^3^)	Surface tension (dyn/cm)	Density (g/cm3)	LogP Value	Lipinski rule validation	Cross Blood brain barrier	Molar refractivity (cm^3^)	Polar surface area (Ǻ^2^) (PSA)	No of HBA	No. of HBD
1	177.8 ± 3.0	23.72 ± 0.5 10–24	53.4 ± 3.0	1.347 ± 0.06	1.52 ± 0.43	Yes	Yes	59.84 ± 0.3	58.2	4	2
2a	244.2 ± 3.0	34.68 ± 0.5 10–24	52.8 ± 3.0	1.288 ± 0.06	2.84 ± 0.44	Yes	Yes	87.48 ± 0.3	58.2	4	2
2b	256.1 ± 3.0	36.62 ± 0.5 10–24	54.0 ± 3.0	1.363 ± 0.06	3.83± 0.46	Yes	Yes	92.38 ± 0.3	58.2	4	2
2c	241.5 ± 3.0	31.63 ± 0.5 10–24	50.7 ± 3.0	1.278 ± 0.06	0.31± 0.58	Yes	Yes	87.89 ± 0.4	58.64	5	1
2d	265.8 ± 5.0	34.84 ± 0.5 10–24	49.3 ± 5.0	1.20 ± 0.1	2.62±0.44	Yes	Yes	87.89 ± 0.4	58.2	4	2
2e	230.7 ± 3.0	33.48 ± 0.5 10–24	58.3 ± 3.0	1.284 ± 0.06	1.51±0.43	Yes	Yes	84.47 ± 0.3	78.43	5	3
2f	268.2 ± 3.0	37.33 ± 0.5 10–24	50.5 ± 3.0	1.285 ± 0.06	2.79± 0.45	Yes	Yes	94.16 ± 0.3	67.43	5	2

FTIR spectral data of compound 1 showed peaks of carboxylic C=O at 1696 cm^–1^ which were missing in the FTIR spectrum of compound 2­(a–f). From this data, the formation of the amide bond in compound 2­(a–f) was quite evident. In compound 1, a singlet of two CH_2_ protons was observed at 4.5 ppm. A doublet of two protons of −CH was observed at 6.95 and 6.10 ppm. Four protons of the aryl group resonated as two doublets at 7.35 and 7.56 ppm. Another singlet of the amide group resonated at 9.73 ppm.

Compound 2a showed a singlet of four protons of benzyl CH_2_ at 3.61 ppm. The doublet of one proton of CH was present at 6.97 ppm, while the doublet of another proton of CH was shown at 7.10 ppm. A multiplet of five protons of the phenyl moiety was observed in the range of 7.26–7.35 ppm. One doublet of two phenyl protons was observed at 7.35 ppm, while the doublet of the other two protons appeared at 7.56 ppm. A singlet of the amide proton was observed at 9.73 ppm.

Compound 2b showed a singlet of two protons of CH_2_ at 3.62 ppm. The doublet of two protons of CH was shown at 6.96 and 7.12 ppm. A multiplet of four phenyl protons was observed in the range of 7.50–7.71 ppm. Two doublets of two Aryl-H appeared at 7.30 and 7.51 ppm. The singlet of a single proton of the amide group resonated at 9.33 ppm

In compound 2c, a multiplet of eight protons of the morpholine group was observed in the range of 3.33–3.52 ppm. A singlet of benzylic CH_2_ protons appeared at 3.61 ppm. Two doublets corresponding to the protons of alkene CH resonated at 6.67 and 7.14 ppm, respectively. The doublet of two protons of the aryl group was present at 7.35 ppm, while the doublet of the other two aryl protons appeared at 7.56 ppm. A singlet of the amide proton was shown at 9.36 ppm.

In compound 2d, a multiplet of ten protons of the cyclohexyl group was observed in the range of 1.11–1.74 ppm. A multiplet of protons of CH appeared at 4.54 ppm. A singlet of two protons of CH_2_ was observed at 3.65 ppm. Two doublets of two protons of CH appeared at 6.66 and 7.2 ppm. Two singlets of two protons of the phenyl group were observed at 7.32 and 7.55 ppm. A singlet of two protons of amide groups was observed at 9.36 ppm.

In compound 2e, a singlet of two protons of CH_2_ was observed at 4.2 ppm. The doublet of two protons of CH appeared at 6.62 ppm, and the doublet of other CH protons resonated at 7.1 ppm. A multiplet of eight phenyl H was shown in the range of 7.2–7.45 ppm. A singlet of one proton of the amide group was resonated at 11.48 ppm.

In compound 2f, a singlet of 3 protons of the methoxy group was observed at 3.8 ppm. A singlet of two protons of CH_2_ group was shown at 4.35 ppm. The doublet of two protons of CH resonated at 6.17 and 7.41 ppm. A multiplet of 8 protons of the aryl group resonated in the range of 6.90–7 ppm. A singlet of amide protons appeared at 9.36 ppm.

### 3.1 Y-Maze Test

Y-Maze test is performed on selected synthetic amide derivatives (2a, 2f) to check the spatial memory by using % spontaneous alternation. Both the compounds have shown an increase in % alternation as shown in Figure 4.

**Figure 4:**
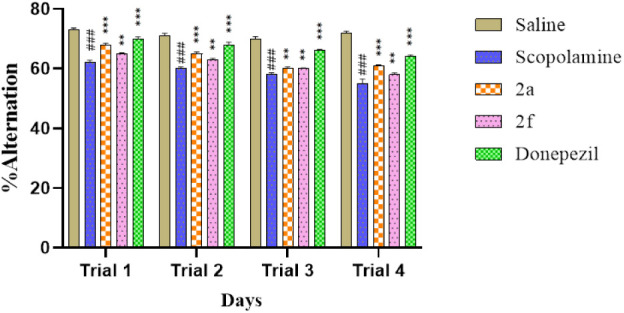
Response of selected synthetic compounds on the percentage of alternation in the Y-Maze test on scopolamine-induced memory deficit rats. Values are expressed as mean ± SEM (*n* = 5). Two way ANOVA. ^###^ *P* < 0.001 vs saline group, *P < 0.05, **P < 0.01, ***P < 0.001 vs scopolamine group.

In days 1, 2, 3, and 4, the % alternation of saline (10 mL/kg) group was 73 ± 0.64, 71 ± 0.83, 70 ± 0.75, and 72 ± 0.51, respectively. % Alternation of the scopolamine group on days 1, 2, 3, and 4 (3 mg/kg) was 62 ± 0.81 (^*###*^ *p* < 0.001 vs saline group), 60 ± 0.63 (^*###*^ *p* < 0.001 vs saline group), 58 ± 1.12 (^*###*^ *p* < 0.001 vs saline group), and 55 ± 1.51 (^*###*^ *p* < 0.001 vs saline group), respectively. % Alternation of the scopolamine +2a (10 mg/kg) group on days 1, 2, 3, and 4 was 68 ± 0.52 (***p < 0.001 vs scopolamine group), 65 ± 0.64 (***p < 0.001 vs scopolamine group), 60 ± 0.63 (**p < 0.01 vs scopolamine group), and 61 ± 0.25 (***p < 0.001 vs scopolamine group), respectively. % Alternation of the scopolamine +2f (10 mg/kg) group on days 1, 2, 3, and 4 was 65 ± 0.36 (**p < 0.01 vs scopolamine group), 63 ± 0.43 (**p < 0.01 vs scopolamine group), 60 ± 0.25 (**p < 0.01 vs scopolamine group), and 58 ± 0.60 (**p < 0.01 vs scopolamine group), respectively. % Alternation of the scopolamine (3 mg/kg) + Donepezil (3 mg/kg) group on days 1, 2, 3, and 4 was 70 ± 0.63 (***p < 0.001 vs scopolamine group), 68 ± 0.85 (***p < 0.001 vs scopolamine group), 66 ± 0.42 (***p < 0.001 vs scopolamine group), and 64 ± 0.67 (***p < 0.001 vs scopolamine group), respectively.

### 3.2 Morris Water Maze Test

Morris water maze test is performed to evaluate the escape time latency in the rats for days 1, 2, 3, and 4. The observed escape latency time is shown in Figure 5.

**Figure 5:**
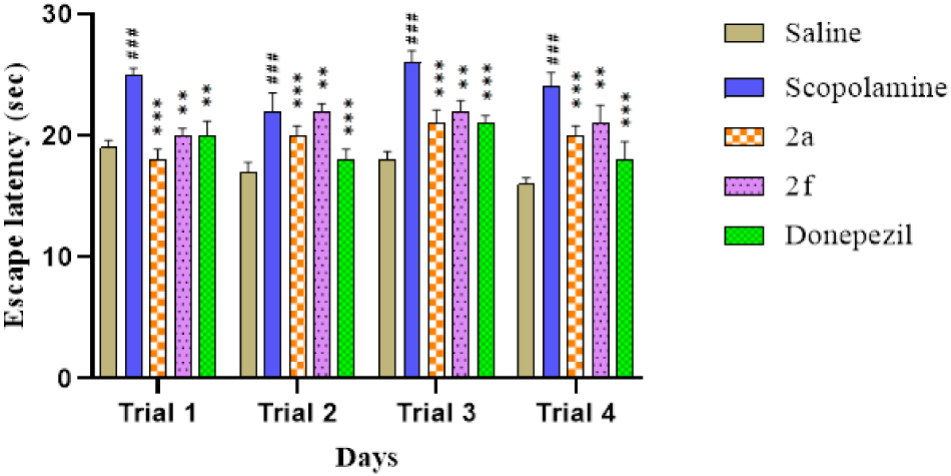
Response of selected synthetic compounds on escape latency of rats in a Morris water maze test trial session to check memory deficit in rats. Values are expressed as mean ± SEM (*n* = 5). Two way ANOVA. ^###^ *P* < 0.001 vs saline group, *P < 0.05, **P < 0.01, ***P < 0.001 vs Scopolamine group.

For the saline (10 mL/kg) group, the values were 19 ± 0.62, 17 ± 0.89, 18 ± 0.74, and 16 ± 0.57, respectively. The latency time of rats on days 1, 2, 3, and 4 of scopolamine (3 mg/kg) administered rats was 25 ± 0.56 (^###^ *p* < 0.001 vs saline group), 22 ± 1.50 (^###^ *p* < 0.001 vs saline group), 26 ± 0.98 (^###^ *p* < 0.001 vs saline group), and 25 ± 1.20 (^###^ *p* < 0.001 vs saline group), respectively. The latency time of scopolamine + compound 2a (10 mg/kg) on days 1, 2, 3, and 4 was observed as 18 ± 0.90 (***p < 0.001 vs scopolamine group), 20 ± 0.79 (***p < 0.001 vs scopolamine group), 21 ± 1.10 (***p < 0.001 vs scopolamine group), and 20 ± 0.80 (***p < 0.001 vs scopolamine group), respectively. The latency time of scopolamine + compound 2f (10 mg/kg) on days 1, 2, 3, and 4 was observed as 20 ± 0.60 (**p < 0.01 vs scopolamine group), 22 ± 0.65 (**p < 0.01 vs scopolamine group), 22 ± 0.90 (**p < 0.01 vs scopolamine group), and 21 ± 1.50 (**p < 0.01 vs scopolamine group), respectively. The latency time of scopolamine + Donepezil (3 mg/kg) on days 1, 2, 3, and 4 was observed as 20 ± 1.20 (**p < 0.01 vs scopolamine group), 18 ± 0.90­(***p < 0.001 vs scopolamine group), 21 ± 0.65 (***p < 0.001 vs scopolamine group), and 18 ± 1.5 (***p < 0.001 vs scopolamine group), respectively.

### 3.3 Free Radical Scavenging Activity by the DPPH Assay

The synthesized compounds have shown marked, comparable antioxidant or free radical scavenging activity in comparison to ascorbic acid as a standard as shown in Figure 6.

**Figure 6:**
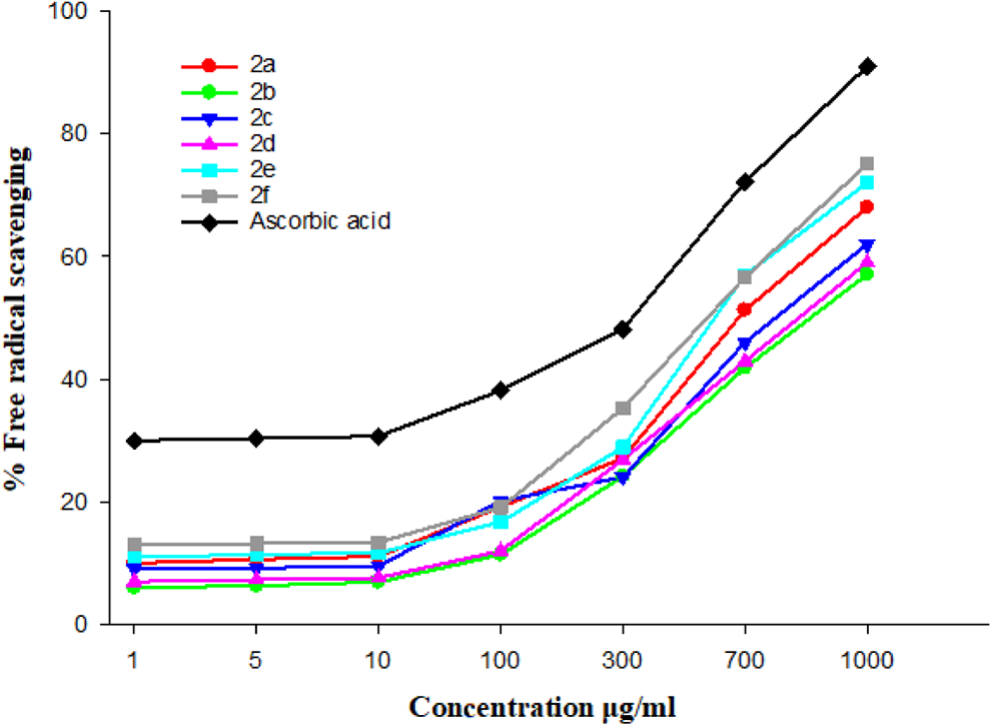
Free radical scavenging activity of different synthetic maleic acid derivatives (2a–2f) in comparison with ascorbic acid by the DPPH assay.

The compounds which show antioxidant activity in the descending order are ascorbic acid < compound 2f < compound 2e < compound 2a < compound 2c < compound 2d < compound 2b.

### 3.4 Acetylcholinesterase (ACE) Inhibition Assay

Following the antioxidant and molecular docking studies, compounds 2a and 2f were further evaluated for their inhibitory effect on ACE. The inhibitory effect of compounds 2a and 2f on ACE was evaluated in comparison with the standard drug Donepezil. Both test compounds demonstrated concentration-dependent inhibition of the enzyme, with compound 2a showing the most significant activity across all tested concentrations (***p < 0.001). In contrast, compound 2f displayed moderate inhibition, which was markedly lower than 2a at reduced concentrations (**p < 0.01). Among the screened molecules, compound 2a emerged as the most potent inhibitor, reflecting a strong correlation with the docking and antioxidant results. These findings suggest that compound 2a holds promise as a lead scaffold for further development of ACE inhibitors. The results of ACE are given in Table 3.

**Table 3:** Inhibitory Effect of the Synthesized Compounds on ACE Activity was Evaluated, and Results are Presented as Mean ± SEM^a^

Compound	Conc. (μg/mL)	% Inhibition (ACE)	IC_50_ values
**2a**	500	85.747 ± 1.34***	20.15
	250	84.840 ± 1.86***	
	125	84.3467 ± 0.86***	
	62.5	77.937 ± 0.49***	
	31.25	61.897 ± 1.47***	
**2f**	500	84.770 ± 2.32***	22.09
	250	80.487 ± 1.72***	
	125	74.590 ± 0.94**	
	62.5	65.293 ± 1.57**	
	31.25	46.553 ± 0.62	
**Donepezil**	500	95.233 ± 0.20	2.82
	250	91.433 ± 0.12	
	125	87.900 ± 0.11	
	62.5	83.167 ± 0.12	
	31.25	77.867 ± 0.08	

a IC_50_ values are presented in nm. Statistical significance was determined in comparison with the reference drug (Donepezil), where *p < 0.05, **p < 0.01, and ***p < 0.001 indicate different levels of significance. Data analysis was performed using two-way ANOVA followed by Tukey’s test.

### 3.5 Immunohistochemical Analysis

In immunohistochemical analysis, the hippocampus and cortex regions are the most affected areas of the brain in Alzheimer’s disease, and the overexpression of COX-2, TNF-α, JNK, and NFκB is quite evident in the scopolamine (Disease) group as compared to the saline group. All the tested compounds have shown marked reduction in the expression of inflammatory markers as compared to the disease group as shown in Figures 7 and 8. While measuring COX-2 response, it is observed that the relative integrated density of COX-2 in the disease group is 0.625 ± 0.213 (^###^ *p* < 0.001 vs saline group), in standard group 3.332 ± 0.265, in 2a group 2.337 ± 0.48 (***p < 0.001 vs disease group), and in 2f group 3.54 ± 0.52. The expression of TNF-α was observed in excessive amount in the disease group 6.661 ± 0.64 (^###^ *p* < 0.001 vs saline group), and in a standard group it is 3.76 ± 0.24 (**p < 0.01 vs disease group). In the compound 2a group, it was 3.979 ± 0.57. In compound 2f, it was 2.557 ± 0.34 (***p < 0.001 vs disease group). The expression of JNK in the disease group was 5.48 ± 0.76 (^###^ *p* < 0.001 vs saline group), and in the standard group it was 3.24 ± 0.36. In compound 2a, it is 2.92 ± 0.55. In compound 2f, it was 2.59 ± 0.45 (***p < 0.001 vs disease group). The expression of NFκB in the disease group was 5.62 ± 0.43 (^###^ *p* < 0.001 vs saline group), in the standard group it was 2.32 ± 0.38, in compound 2a it was 2.05 ± 0.38 (***p < 0.001 vs disease group), and in compound 2f, it was 2.12 ± 0.43 (**p < 0.01 vs disease group).

**Figure 7:**
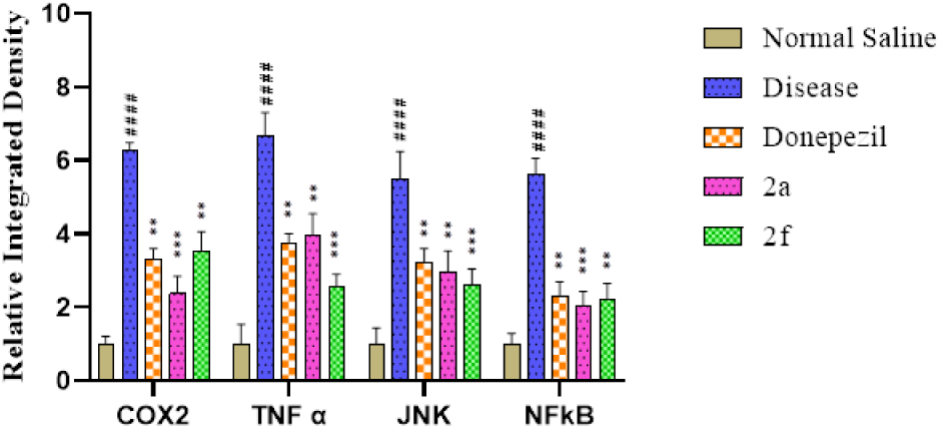
Relative integrated density of COX-2, TNF α, JNK, and NFκB expression in rat brain tissues. Values are expressed as mean ± SEM (*n* = 5). Two way ANOVA. ^###^ *P* < 0.001 vs saline group, *P < 0.05, **P < 0.01, ***P < 0.001 vs scopolamine group.

**Figure 8:**
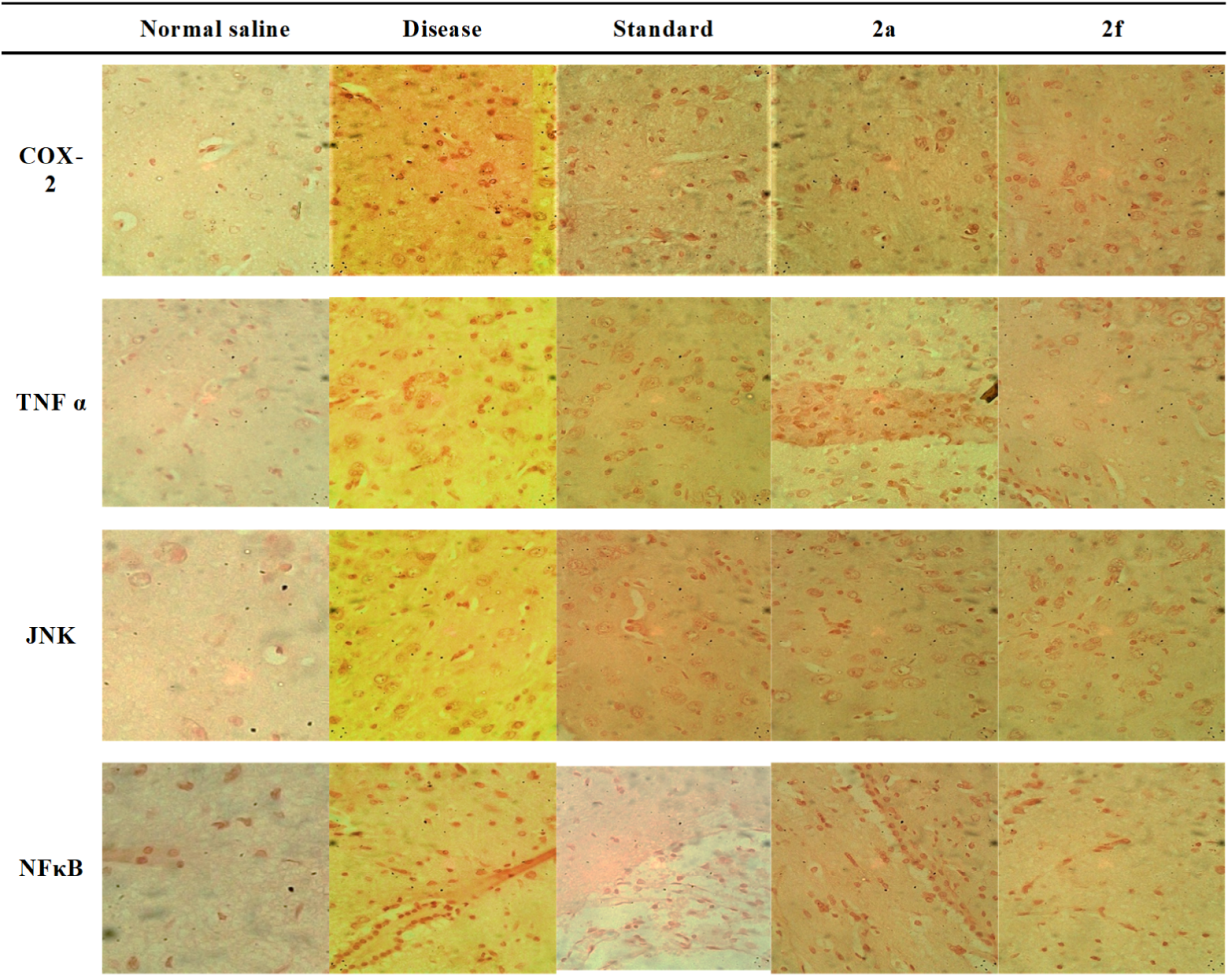
Immunohistological representation of rat brain tissues.

### 3.6 Hematoxylin and Eosin Staining

Hematoxylin and eosin staining was used in the evaluation of brain tissues in the cortex region. The hippocampus and cortex regions are the most affected areas of brain in Alzheimer’s disease. Results are shown in Figure 9.

**Figure 9:**
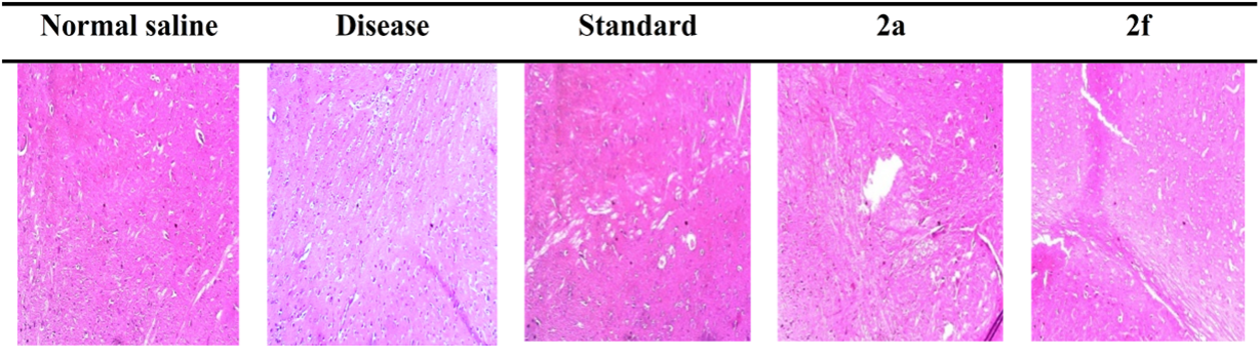
Histopathological visualization of the cortex area of rat brain tissue.

That in the rat treated with normal saline (10 mL/kg), the cellular structure of the brain tissue is intact, and the normal appearance of the tissue was evident, whereas the diseased group which was given scopolamine (3 mg/kg) showed an abnormal cellular morphology. The cellular structure was disrupted, showing damage in the cortex region, which is responsible for the abnormal functionality of the brain. The group with the standard treatment, Donepezil (3 mg/kg), and treatment with test compounds 2a (10 mg/kg) and 2f (10 mg/kg) showed marked improvement in the cellular structure of the brain tissue. This indicated that the standard and test compounds have a similar neuronal protective function, which was quite evident from the slides.

### 3.7 Molecular Docking

Docking of the ligand with target proteins, COX-2 (PDB-ID 5F19), TNFα (PDB-ID 5MU8), JNK (PDB-ID 2G01), NFκB (PDB-ID 1SVC), GSK-3β (PDB-ID 1Q3W), and ACE (PDB-ID 1ACJ) shows the highest binding affinity of the synthesized molecules with the target proteins, as given in Table 4. Additionally, 3D binding interaction visualizations are shown in Figure 10.

**Table 4:** Binding Affinities of Compounds (2a–2f) with COX-2 (5F19), TNFα (5MU8), JNK (2G01), NFκB (1SVC) GSK-3β (1Q3W), and ACE (1ACJ)

Ligand	Binding affinity(Kcal/mol)COX-2	Binding affinity(Kcal/mol)TNFα	Binding affinity(Kcal/mol)JNK	Binding affinity(Kcal/mol)NFκB	Binding affinity(Kcal/mol)GSK-3β	Binding affinity(Kcal/mol)ACE
**2a**	–8.4	–7	–7.1	–7	–7.4	–10.3
**2b**	–8.2	–6.9	–7.3	–6.4	–7.7	–10
**2c**	–8.1	–6.7	–7.2	–6.1	–7.4	–7.3
**2d**	–8.1	–6.9	–6.4	–6.5	–7.5	–10.2
**2e**	–8.1	–6.9	–6.7	–6.9	–7.6	–8.8
**2f**	–8.6	–6.6	–6.3	–6.9	–7.5	–7.4

**Figure 10:**
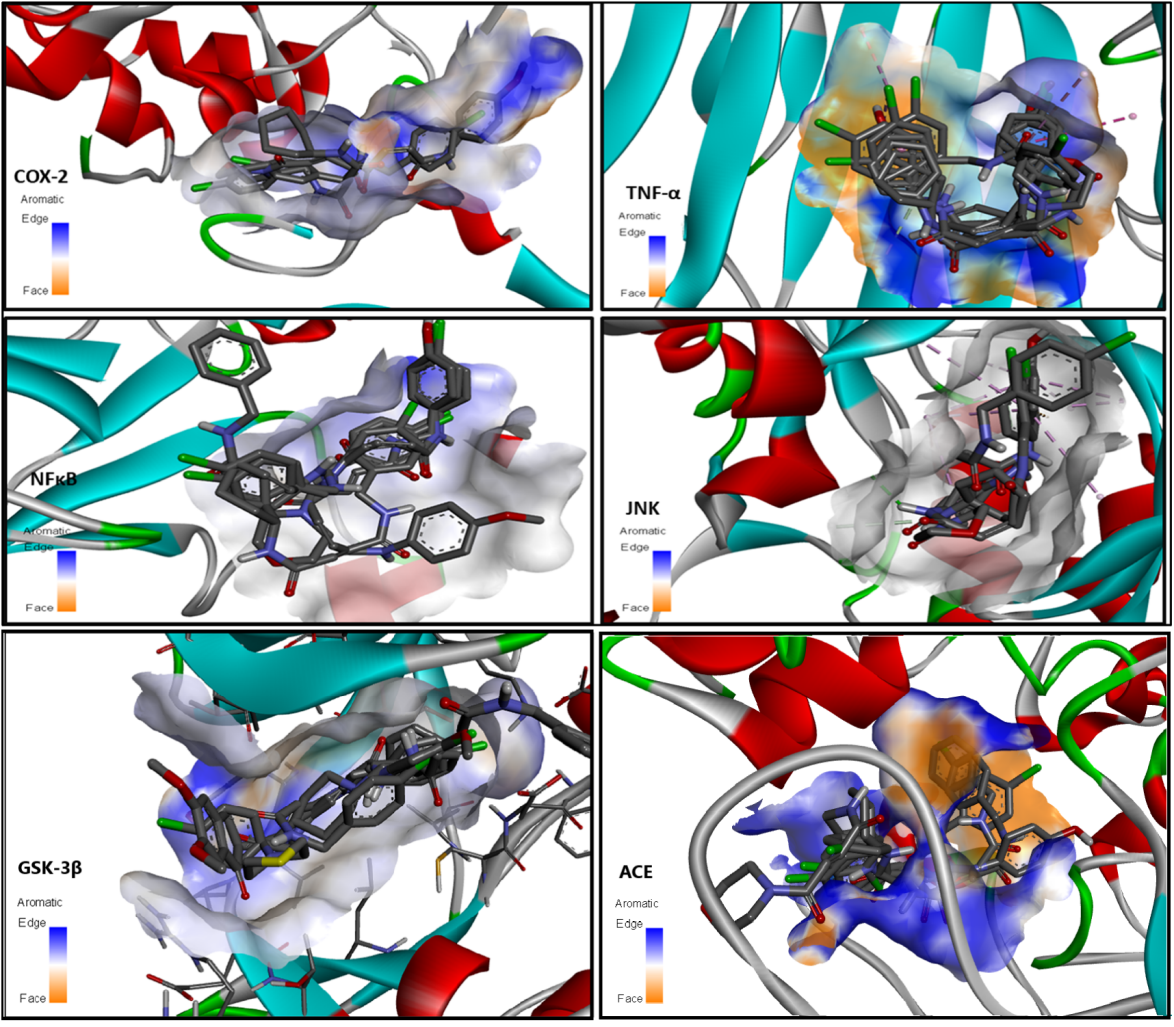
Docking 3D visualization of synthesized compounds with target proteins (COX-2, TNF-α, JNK, NFκB, GSK-3β, and ACE).

### 3.8 Effect of the Derivatives (2a and 2f) on Oxidative Stress and Neuroinflammation

Administration of scopolamine markedly induced oxidative stress, as evidenced by a decline in antioxidant enzymes (GSH, GST, and catalase) along with an increase in lipid peroxidation. Treatment with the synthesized derivatives 2a and 2f significantly restored the enzymatic antioxidant defense, showing higher levels of GSH, GST, and catalase compared to the disease group, while also reducing lipid peroxidation toward near-control levels. Between the two, compound 2a demonstrated slightly superior antioxidant protection, though both derivatives produced effects comparable to the standard treatment as shown in Table 5. These findings indicate that 2a and 2f effectively counteract scopolamine-induced oxidative stress, thereby reducing neuronal vulnerability and neuroinflammation associated with Alzheimer’s pathology.

**Table 5:** All Values are Presented as the Mean ± SEM (*n* = 5 per Group)^a^

Groups	GSH (μmol/mg of protein)	GST (μmoles CDNB conjugate/min/mg of protein)	CAT (Catalase) (μmoles H202/min/mg of protein)	LPO (Tbras-nM/min/mg protein)
Control	10.33 ± 0.61	1.97 ± 0.034	23.37 ± 5.61	28.143
Standard	7.12 ± 0.17	1.32 ± 0.041	21.43 ± 3.15	28.710
Disease	4.09 ± 0.06	0.69 ± 0.59	15.66 ± 3.42	39.181
2a	6.45 ± 0.13	1.16 ± 0.018	19.41 ± 2.93	27.981
2f	6.99 ± 0.21	1.09 ± 0.021	18.51 ± 2.11	27.132

a Statistical significance was evaluated relative to the control and disease groups. Symbols * and ** indicate significance at P < 0.05 and P < 0.01, respectively, while *** denotes highly significant differences at P < 0.001. Similarly, #, ##, and ### represent differences relative to the disease group at P < 0.05, P < 0.01, and P < 0.001, respectively. Abbreviations: GSH, glutathione; GST, glutathione-S-transferase; CAT, catalase; LPO, lipid peroxidation; TBARS, thiobarbituric acid reactive substances.

## 4 Conclusions

In this study, novel 4-chlorobenzylamine-containing maleic acid derivatives were successfully synthesized, characterized, and evaluated for their potential anti-Alzheimer’s activity. The purity of the synthesized compounds was confirmed using TLC, while their structural characterization was performed using FTIR ^1^H NMR and ^13^C NMR spectroscopy. FTIR analysis confirmed the formation of amide bonds, and NMR data provided insights into the chemical environment of the synthesized compounds.

Among the synthesized derivatives, compound 2a exhibited the highest binding affinity (−10.3 kcal/mol, −7.0 kcal/mol, and −7.1 kcal/mol) for ACE, TNFα, and NFκB, respectively, while compound 2b showed the highest affinity (−7.3 kcal/mol) for JNK, indicating their potential as anti-inflammatory agents. Although compound 2b showed strong interaction with JNK, compound 2f exhibited better overall antioxidant potential and a favorable interaction profile across other targets, such as antioxidant activity in the DPPH assay, highlighting its potential neuroprotective effects. Furthermore, the comparative analysis of compounds 2a and 2f revealed that 2a exhibited greater potency and superior inhibitory activity against ACE.

The in vivo studies further validated the neuroprotective potential of the test compounds. Behavioral assessments, including the Y-maze test and Morris water maze test, indicated improved spatial memory and cognitive performance, with compound 2f showing superior activity. Immunohistochemical and histopathological analyses revealed a significant reduction in inflammatory markers (COX-2, TNFα, JNK, and NFκB) and an increase in neuronal survival, suggesting a neuroprotective effect comparable to the standard treatment (Donepezil). The *in vivo* antioxidant assays demonstrated that compound 2a provided stronger protection by enhancing catalase and GST activity, while compound 2f showed better improvement in GSH levels. Both compounds also reduced lipid peroxidation which confirm their neuroprotective potential comparable to the standard drug.

The study overall demonstrates that the synthesized maleic acid derivatives, particularly compounds 2a and 2f, have promising anti-Alzheimer potential due to their strong binding affinities, anti-inflammatory properties, antioxidant activity, and neuroprotective effects. Their lipophilic nature allows them to cross the blood–brain barrier, making them strong candidates for further development as therapeutic agents for Alzheimer’s disease. Future studies involving detailed pharmacokinetics, toxicity profiling, and amyloid-β or tau quantification are warranted to advance these compounds toward clinical applications.

## Data Availability

The materials supporting the findings of this study are available from the corresponding author upon request.
